# Metabolic profiling of a polycystic ovary syndrome-like organoid model reveals the critical role of glutamine in local endometrial dysregulation related to implantation failure

**DOI:** 10.3389/fcell.2026.1751258

**Published:** 2026-03-25

**Authors:** Haoxuan Yang, Jing Zhang, Su Long, Yuhuan Xue, Jinfeng Tan, Ge Chen, Yongqi Luo, Ricardo Azziz, Chichiu Wang, Wenming Xu, Xiaomiao Zhao

**Affiliations:** 1 Department of Obstetrics and Gynecology, Key Laboratory of Obstetric, Gynecologic and Pediatric Diseases and Birth Defects of Ministry of Education, West China Second University Hospital, Sichuan University, Chengdu, Sichuan, China; 2 Department of Reproductive Medicine, Guangdong Provincial People’s Hospital (Guangdong Academy of Medical Sciences), Southern Medical University, Guangzhou, Guangdong, China; 3 Fisheries College, Hunan Agricultural University, Changsha, Hunan, China; 4 Department of Obstetrics and Gynecology, The First Affiliated Hospital, Sun Yat-sen University, Guangzhou, Guangdong, China; 5 Southern Medical University, Guangzhou, Guangdong, China; 6 Department of Health Policy, Management, and Behavior, School of Public Health, University at Albany, SUNY, Rensselaer, NY, United States; 7 Chinese University of Hong Kong-Sichuan University Joint Laboratory in Reproductive Medicine, The Chinese University of Hong Kong, Hong KongSAR, China

**Keywords:** endometrial organoids (EOs), extra-organoid fluid (EOF), intra-organoid fluid (IOF), metabolomics, polycystic ovary syndrome (PCOS)

## Abstract

Polycystic ovary syndrome (PCOS) is a prevalent endocrine disorder characterized by reproductive and metabolic disturbances, which causes a chronic lack of ovulation that leads to increased incidence of atypical endometrial hyperplasia and carcinogenesis. Increasing evidence indicates that metabolic changes may play a crucial role in PCOS pathogenesis; however, the metabolic profile of fluid in PCOS-related endometrium has not yet been characterized. In this study, we successfully constructed three cases of endometrial organoids derived from clinically healthy endometrium. We established a high-androgen model by adding different ratios of estradiol and testosterone to simulate PCOS-like characteristics. Through scanning electron microscopy and immunofluorescence detection, we found that extra androgen treatment-induced cellular damage led to cellular fragments and apoptosis. The intra-organoid fluid (IOF) and extra-organoid fluid (EOF) of the organoids were separated and analyzed by high-throughput quantitative metabolomics. The results showed that amino acid metabolism, specifically glutamine metabolic changes, was the major metabolic pathway altered in the EOF; meanwhile, changes in fatty acids were the main metabolites in the IOF among the groups. Specifically, the *in vitro* model confirmed that glutamine enhances endometrial stromal cell decidualization with altered mitochondrial function during the implantation process, which may provide the basis for metabolic marker screening and for identifying potential metabolic targets for intervention in female infertility related to PCOS.

## 1 Introduction

Polycystic ovary syndrome (PCOS) is a complex disorder characterized by a diverse interplay of endocrine, reproductive, and metabolic features (Escobar-Morreale, 2018; Helvaci and Yildiz, 2025). Globally, it is estimated to affect approximately 6%–20% of women. According to the Rotterdam criteria (Aflatounian et al., 2020), a diagnosis of PCOS typically requires the presence of hyperandrogenism along with insulin resistance (Teede et al., 2018). Hyperandrogenism constitutes a fundamental abnormality in PCOS, causing disruptions in steroidogenesis that impair ovulation, ultimately leading to the development of polycystic ovaries. Although primarily recognized as a reproductive endocrine disorder, PCOS is commonly associated with additional manifestations such as obesity, the already mentioned insulin resistance, and impaired glucose tolerance. These metabolic disturbances can predispose individuals to serious health issues, including cardiovascular disease and type 2 diabetes (Shrivastava and Conigliaro, 2023). Collectively, the multiple symptoms associated with PCOS frequently lead to infertility, including implantation failure.

Beyond inducing ovarian irregularities, PCOS significantly impacts the endometrium—a tissue primarily regulated by steroid hormones. Emerging research indicates that PCOS disrupts the formation of the endometrial microenvironment, primarily through mechanisms associated with chronic inflammation. This inflammatory response is believed to be driven by the overexpression of androgen receptors (ARs) and Toll-like receptors (Hu et al., 2021), which trigger a robust immune response. During the window of implantation, immune cells, including natural killer (NK) cells (Gamliel et al., 2018), regulatory T cells (Tregs) (Huang, Chi, and Qiao, 2020), and effector T cells (Teffs) (Mahajan et al., 2023), are typically enriched in the decidua. However, patients with PCOS have significant alterations in this immune landscape, such as increased NK cells (He et al., 2020) and reduced Tregs (Krishna et al., 2015). Additionally, studies have shown substantial alterations in metabolites associated with the tricarboxylic acid (TCA) cycle within the endometrium of individuals with PCOS. These metabolic changes are believed to contribute to immune cell activation and heightened inflammatory states, which may ultimately impair embryo implantation (Chen et al., 2023). Although current research provides valuable insights into these processes, many studies rely on endometrial biopsies for evaluation, which is a minimally invasive procedure with low risk; however, it is also limited in its ability to reflect dynamic metabolic changes during the menstrual cycle. Moreover, little attention has been given to changes in the luminal microenvironment, specifically. Considering the presence of more pathogenic clues, it is imperative to develop a continuous dynamic observation model to analyze the metabolic alterations within the endometrial luminal microenvironment. Understanding these localized metabolic changes could advance our knowledge of PCOS pathogenesis and facilitate the development of personalized diagnostic and therapeutic strategies that are tailored to the specific metabolomic profiles of the affected individuals.

The composition of the uterine fluid (UF) undergoes significant changes that are closely associated with pregnancy loss (Simintiras et al., 2021). For successful embryo adhesion, the liquid present on the surface of the endometrium is minimal (Ruan, Chen, and Chan, 2014), making it challenging to detect changes in metabolites. Endometrial organoids (EOs), which are filled with internal fluid, have emerged as promising model systems for studying the cellular and molecular mechanisms associated with endometrial biology, pregnancy-related disorders, endometriosis, endometrial hyperplasia, and endometrial cancer (EC) (Boretto et al., 2019). Recent scaffolding-free multicellular EOs have revealed that excessive androgen levels in PCOS promote cell proliferation and lead to the dysregulation of genes associated with cell proliferation and migration. This discovery has uncovered a novel mechanism contributing to the increased risk of EC in individuals with PCOS (Wiwatpanit et al., 2020). Furthermore, comparative analyses of the metabolomic profiles and RNA sequencing between extra-organoid fluid (EOF) and intra-organoid fluid (IOF) have highlighted donor-specific biochemical signatures in organoids. These findings indicate that transcriptomic regulation likely underpins the observed secretory asymmetry, providing a foundational understanding of UF composition and its regulation, with potential implications for female fertility and offspring health (Simintiras et al., 2021). However, no studies have yet explored the differences between uterine fluid and external fluid metabolism in a high-androgen hormone environment in PCOS patients, a subject that warrants further investigation.

In the present study, we successfully established three examples of EOs using clinical endometrial biopsies from three female patients. These organoids were later recognized and verified using morphological analysis and immunofluorescence experiments. We created EOs that resembled the PCOS-like hormonal environment by varying the amounts of estrogen and androgen. The organoids' morphology was examined using TEM to determine the effects of high androgen levels, and the expression of the estrogen receptors (ERs) and ARs was examined using immunofluorescence. The IOF and EOF of the organoids were separated and sequenced by high-throughput quantitative metabolomics, and the differences in metabolomics between the IOF and EOF of the PCOS-like organoids were analyzed. The variations in amino acid metabolism and their impact on energy metabolites were further examined using cell culture and *in vitro* implantation experiments, which may provide the basis for metabolic marker screening and potential metabolic targets for intervening in female infertility related to PCOS.

## 2 Materials and methods

### 2.1 Human endometrial organoid culture

Endometrial biopsies were collected from hormonally untreated patients undergoing laparoscopy for benign gynecological conditions at Guangdong Provincial People’s Hospital. The study received approval from the hospital’s Ethical Committee (KY-Q-2022–369-01), and written informed consent was obtained from all participants. The donors’ biopsies and related details are listed in Supplementary Table S1.

In this study, the protocol for organoid establishment referred to earlier studies (Yang et al., 2025; Fitzgerald et al., 2019; Turco et al., 2017). In brief, after laparoscopy, the endometrial samples were placed in precooled PBS (Biosharp, China) supplemented with a 10% penicillin–streptomycin solution (Gibco, Grand Island, United States) and transported to the laboratory for further processing. The tissue samples were washed thrice in precooled 0.01% benzyldodecyldimethylammonium bromide (Macklin, China) and precooled PBS with 1% penicillin–streptomycin solution and gently stirred to remove the contaminating blood. The tissue sample was then minced into 0.5 mm^3^–1 mm^3^ pieces in a Petri dish containing PBS. After centrifugation at 1,000 rpm for 10 min, the supernatant was discarded. An appropriate amount of precooled 10% collagenase I (HARVEYBIO, China) was added, and the samples were incubated at 37 C for 40 min–50 min to ensure complete digestion. Digestion was terminated by adding an Advanced DMEM/F12 medium (Thermo Fisher Scientific, Grand Island, United States) with fetal bovine serum (FBS, Gibco, Grand Island, United States). The precipitate was collected through centrifugation at 1,000 rpm for 10 min. The obtained mixture was passed through 70-μm and 40-μm cell sieves sequentially, and the glandular elements were resuspended in a mixture of 70% Matrigel (Coring, Bedford, MA, United States) and 30% Advanced DMEM/F12. Approximately 20 μL of the mixture were deposited in 48-well plates (Coring, Bedford, MA, United States), which were then set at 37 °C and overlaid with approximately 250 µL of organoid expansion medium (ExM). The medium was changed every 2–3 days, and the organoids were passaged every 7–10 days. Once the Matrigel was removed, the organoids were digested with TrypLE Express (Thermo Fisher Scientific, Grand Island, United States) and resuspended in Recovery cell culture freezing medium (Thermo Fisher Scientific, Grand Island, United States) for freezing. The components of the ExM and the related reagents are provided in Supplementary Table S2. Organoids with low passage numbers (P3−P6) were utilized for the experiments described.

### 2.2 Immunofluorescence and TUNEL analysis

After three passages of organoid culture, 2–3 wells of organoids mixed with PBS were collected. The selected organoids for analysis had the following characteristics: 1) intact shape, 2) fewer dead cells in the chamber, and 3) plenty and bright cytoplasm. These characteristics ensured that the organoids were in a healthy state. In addition, to avoid the influence of the passage, organoids were used in the trial with different passages to ensure the findings were consistent. The organoids were pipetted up and down multiple times to break them apart. The resulting suspension was resuspended in 1 mL of 4% paraformaldehyde (PFA, Biosharp, China) and centrifuged at 600 rpm for 5 min. Some of the fixed organoids were sent to Guangzhou Tenngbio for ultracryotomy, while others were prepared for immunofluorescence. Thin sections (∼10 µm) were loaded onto polylysine slides. The fixed organoids were permeabilized for 15 min in 0.01% Triton X-100 in PBS and subsequently blocked with 4% bovine serum albumin (BSA, Macklin, China) in PBST buffer (Macklin, China) at room temperature for 2 h. The samples were then incubated overnight at 4 °C with primary antibodies, including CK18, E-cadherin, MUC1, vimentin, AR, ER, CDX2, TOMM20, COX2, and OPA1. All details of the antibodies are listed in Supplementary Table S3. After washing with PBST, the samples were incubated with anti-mouse IgG-Alexa 488 (1:1,000 dilution) and anti-rabbit IgG-Alexa 594 (1:1,000 dilution) (Thermo Fisher Scientific, CA, United States). After washing again with PBST, the organoids and the sections on the slides were incubated with DAPI solution (0.25 mg/mL) at 26 °C for 15 min. After mounting with coverslips using an anti-fade mounting medium (Invitrogen, CA, United States), the color of the antibody staining in the tissue sections was observed under an inverted fluorescence microscope (Nikon TI-S, Japan).

For TUNEL staining, we used the TUNEL kit (A113, Vazyme, China). The section was permeabilized by proteinase K for 30 min after dewaxing and then washed thrice in PBS. After pretreatment, the section was set in the equilibration buffer at room temperature for 30 min, labeled with BrightRed Labeling Mix for 60 min at 37 °C, and then re-stained with DAPI. Pictures were captured using a SpinSR10 (Olympus, Japan).

### 2.3 Hormone treatment

Considering the complex pathological factors of PCOS, we simplified the *in vitro* test model and adapted extra androgen induction to detect the metabolic changes in the endometrial luminal microenvironment after additional androgen treatment. Three different hormone treatment groups were constructed according to the previous protocol (Wiwatpanit et al., 2020):

1. Control group: no hormonal treatment was administered.
2. Normal group: treated with 0.1 nM estradiol (E2, Sigma, Germany) and 0.8 nM testosterone (T, Hkhcbio, China) on days 0–7, treated with 1 nM E2 and 0.8 nM T on days 7–13, and treated with 1 nM E2 and 1.25 nM T on days 13–14.
3. PCOS-like group: treated with 0.1 nM E2 and 3 nM T on days 0–7, and treated with 1 nM E2 and 3 nM T on days 7–14.

The corresponding media were changed every 2 days. After treatment, both the hormone-treated and control organoids were fixed for immunofluorescence and prepared for transmission electron microscopy (TEM) analysis following the previously described procedures.

### 2.4 TEM analysis

Three organoids from the different groups were fixed overnight in 2.5% glutaraldehyde (Sigma, St. Louis, MO, United States) in PBS buffer at 4 °C. The fixed organoids were centrifuged (200 ×g), pelleted, and washed thrice in PBS. The pellets were resuspended in 1.5% low-melting-point agarose and pelleted (1000 ×g). After solidification, the pellets were cut into small cubes, washed with PBS, and post-fixed in 1% osmium tetroxide (SPI, China). The samples were then washed again and dehydrated using graded ethanol (50%, 70%, 80%, 90%, and 100%; 10 min–15 min for each grade and repeated twice) and acetone. After soaking in acetone overnight, the samples were embedded. The embedded samples were polymerized at 60 °C for 2 days. Thin 70-nm sections were cut with a Leica UC7 (Leica, Germany) ultramicrotome and analyzed using the Tecnai G2 Spirit transmission electron microscope (FEI Company, Hillsboro, Oregon, United States).

### 2.5 Extraction of extra-organoid fluid (EOF) and intra-organoid fluid (IOF) from endometrial epithelial cell organoids

The EOF and IOF were extracted from the hormone-treated and control groups according to the methods described in a previous publication (Simintiras et al., 2021). In brief, total EEO-conditioned ExM (EOF) was aspirated from each well, pooled by donor, snap-frozen in liquid nitrogen (N_2_(l)), and stored at −80 °C until analysis. The IOF was then separated by high-throughput centrifugation (HTC), as described below. The medium was sucked from the organoid culture hole and replaced twice with precooled PBS. The organoids were then incubated with chilled Cell Recovery Solution (Coring, Bedford, MA, United States) at 4 °C for 30 min. A 1-mL microtube attached to the tip of the wide hole was used to gently transfer the organoids to a 15-mL tube and centrifuged for 10 min at 4 °C at 270 × g. Then, the supernatant was removed, and the microsphere was set in 3 mL freezing 1x PBS, centrifuged, and resuspended in 250 μL of freezing 1x PBS again. The organoids were then transferred to a 1.5-mL tube, rotated slightly for 5 min, and then centrifuged at 4 °C 3,750 × g for 15 min. Diluted IOF (upper clear layer) was transferred to a 1.5-mL tube, frozen quickly in N_2_ (L), and stored at −80 °C for analysis.

### 2.6 Q300 targeted metabolomic array quantification

This trial followed the study by Xie et al. (2021); targeted metabolomic sequencing (Q300™ Total Quantitative Metabolomics) of the EOF and IOF was carried out by the Shanghai Wayen Biotechnologies company, the website is: https://www.wayenbio.com/intro/14.html. This project uses a strict quality-control/assurance procedure to ensure the highest quality samples from receipt to delivery. Every step of the final delivery process ensures consistently high-quality analytical results. In brief, to reduce degradation, the samples were thawed in an ice bath, and 20 μL of each sample was added to a 96-well plate. The plate was transferred to the Eppendorf epMotion Workstation (Eppendorf Inc., Germany). Adding the pre-cooled 120 µL methanol solution (Sigma, St. Louis, MO, United States) and swirl violently for 5 min, then 4000 ×g, 30 min at 4 °C. Returning the plate to the workstation, 20 μL of freshly prepared derivatives were added to each well, and the plate was sealed and placed at 30 °C for 60 min for derivatization. Then, 330 μL of precooled 50% methanol solution was added to the plate, followed by centrifugation at 4 °C (4,000 ×g, 30min). Next, 135 μL of the supernatant was transferred to a new 96-well plate, and 10 μL of the internal standard was added to each well; then, the gradient diluent of the biochemical standard stock solution was added to the left hole and sealed for LC-MS analysis (UPLC-MS/MS, ACQUITYUPLC-Xevo TQ-S, Milford, MA, United States). Data acquisition and metabolite quantification were performed using Targeted Metabolome Batch Quantification (TMBQ) software (v1.0, HMI, Shenzhen, Guangdong, China). There are six functional modules in the main workflow of TMBQ, including raw data reading and pretreatment, peak picking, intensity calculation and correction, standard curve fitting, concentration calculation, and results generation and exportation.

The raw data files generated by UPLC-MS/MS were processed using the MassLynx software (version 4.1, Waters, Milford, MA, USA). For each metabolite, peak integration, calibration, and quantification were performed, and the iMAP platform (v1.0, Metabo-Profile, Shanghai, China) was used for statistical analysis. If the data of a certain metabolite in all the groups followed a normal distribution (Shapiro test P > 0.05) and had homogeneity of variance (Levene’s test P > 0.05), then parametric test methods were used for this metabolite. A t-test was used for comparisons between two groups; a one-way analysis of variance (ANOVA) test was used for comparisons among multiple groups. The *post hoc* test used was Tukey’s test, and the fold change (FC) between groups was calculated using the ratio of means.

Otherwise, nonparametric test methods were used. The Wilcoxon test was used for comparisons between two groups; the Kruskal–Wallis test was used for comparisons among multiple groups. The *post hoc* test used was Dunn’s test, and the fold change (FC) between groups was calculated using the ratio of medians.

Mass spectrometry-based quantitative metabolomics involves determining the concentration of an unknown substance by comparing it to a set of standard samples with known concentrations (i.e., a calibration curve). A calibration curve is a graph showing the variation of the analytical signal with the concentration of the analyte (i.e., the substance being analyzed). For the majority of analyses, the instrument response is linearly related to the concentration, resulting in a model presented by the equation y = ax + b, where y is the instrument response (such as peak height or area), a is the slope/sensitivity, and b is the constant describing the background. The concentration (x) of the analyte in the unknown sample can be calculated using this equation.

A series of operations for data processing, interpretation, and visualization is carried out using the iMAP platform. In metabolomics research, two statistical analysis methods are widely used: 1) multivariate statistical analysis, such as principal component analysis (PCA), partial least squares discriminant analysis (PLS-DA), orthogonal partial least squares discriminant analysis (OPLS-DA), random forest method, and support vector machine learning. 2) Univariate statistical analysis, including the t-test, Mann–Whitney–Wilcoxon (U-test), analysis of variance, and correlation analysis. The best choice of statistical method usually depends on the data and project goals.

### 2.7 Cell culture and attachment experiment

Aiming to verify the metabolomic result that the PCOS-like group showed an increase in glutamine metabolic activity that may affect the decidual process, we used human endometrial stromal cells (hESCs) derived from healthy individuals’ endometrial biopsies for *in vitro* decidualization to detect the effect with different concentrations of glutamine. hESCs were cultured in a Petri dish with phenol-free DMEM/F12 (Gibco, Grand Island, United States) supplemented with charcoal-stripped fetal bovine serum (BI, IL), and the cell type was confirmed by immunofluorescence. Then, the cells were stimulated with 10 nM E2 (Sigma, St. Louis, MO, United States) + 1 μM P4 (Sigma, St. Louis, MO, United States) + 1 μM cAMP (Sigma, St. Louis, MO, United States) + glutamine (0, 2, and 4 mM) after the cells were 80% aggregated, and the cell culture without hormones was set as a control group. To verify that epithelial glutamine metabolism can affect the decidualization of stromal cells, we used a conditional culture medium that organoid D5 culture medium which was used to induce secretory changes. Decidual levels were detected by qPCR and immunofluorescence.

To analyze the glutamine function in embryo implantation, we used a 2D co-culture model containing Ishikawa cells (Procell, China) and human trophoblast stem cells (hTSCs) (Li et al., 2024) (donated by the Peng Du laboratory at Peking University). The Ishikawa cells were cultured in a Petri dish with phenol-free DMEM/F12 (Gibco, Grand Island, United States) supplemented with charcoal-stripped fetal bovine serum (BI, IL) and then stimulated with 10 nM E2 (Sigma, St. Louis, MO, United States) + 1 μM P4 (Sigma, St. Louis, MO, United States) + 1 μM cAMP (Sigma, St. Louis, MO, United States) to transition to the secretory stage, which served as the positive control, after the cells were 80% aggregated. Based on fundamental hormone stimulation, we added glutamine (0, 2, and 4 mM) to the treated group, and the cell culture without hormone treatment was set as the negative control group.

The hTSCs were mixed with Matrigel at a concentration of 10^4^ cells per 1 mL; a 30 μL drop of the mixture was added to a Petri dish, and the cells were covered with culture medium after solidification. After 4 days, these cells formed a sphere in the Matrigel. Then, the Matrigel drops were washed with ice-cold PBS and collected in a 1.5-mL centrifuge tube; these mixtures were placed on ice until the Matrigel melted. After centrifugation, the supernatant was discarded, and the spheres were resuspended in fresh culture medium containing CellTracker Red (MCE, New Jersey, United States), while the single cell layer was stained with CellTracker Blue (MCE, New Jersey, United States), and washed after 45 min of incubation. Then, using an open-pull straw, we added 20 spheres to the cell layer and measured the adhesion rate after 24 h based on the previous literature (Aberkane et al., 2018).

For adhesion rate analysis, we captured the initial figure after 24 h and then used a pipette to gently wash the implanted hTSC spheres thrice with warmed culture medium and captured the figure again. We divided the number of hTSC spheres still attached by 20 to obtain the final adhesion rate, and we calculated the area of trophoblast cell invasion.

### 2.8 RT-qPCR

For relative mRNA expression analysis, we performed a real-time RT-qPCR assay using a CFX 96TM Real-Time PCR Detection System (Bio-Rad, Hercules, CA, USA). A real-time RT-qPCR assay was performed using a Takara RR820A kit (Takara, Japan) according to the manufacturer’s instructions. For this purpose, we used a 10-μL reaction mixture containing 1 μL of cDNA, 5 μL of TB Green Premix Ex Taq II (Takara, Kusatsu, Shiga Prefecture, Japan), 0.5 μL of each of the forward and reverse primers, and 3 μL of ddH_2_O. Glyceraldehyde 3-phosphate dehydrogenase (*GAPDH*) was used as an internal reference gene for normalization. The PCR conditions were as follows: an initial denaturation at 95 °C for 3 min, followed by 40 cycles of denaturation at 95 °C for 10 s, annealing at a primer-specific temperature for 30 s, and extension at 72 °C for 30 s. A melting curve analysis was performed to validate the specificity of the reaction. Only one product of the desired size was identified, and a single smooth peak was observed for each primer in the melting curve analysis. Each sample was analyzed in triplicate. The relative mRNA expression levels were calculated using the 2^−ΔΔCT^ (Livak) method. All the primers used are listed in Supplementary Table S4.

### 2.9 Statistical analysis

MassLynx software (v4.1, USA) was used to process the original data files obtained from UPLC-MS/MS for integrating, calibrating, and quantifying each metabolite. The iMAP platform was used for statistical analysis. Data presentation adheres to the format of mean ± standard deviation (SD). Statistical evaluations were conducted using GraphPad Prism (GraphPad Software, San Diego, California, United States). For the assessment of differences between the two groups, two-tailed Student's t-tests were implemented. ANOVA was utilized when comparing more than two groups, with Dunnett’s test serving for subsequent group-to-group comparisons. In the graph, * indicates P < 0.05, ** indicates P < 0.01, and *** indicates P < 0.001.

## 3 Results

### 3.1 Establishment of endometrial organoids

**FIGURE 1:**
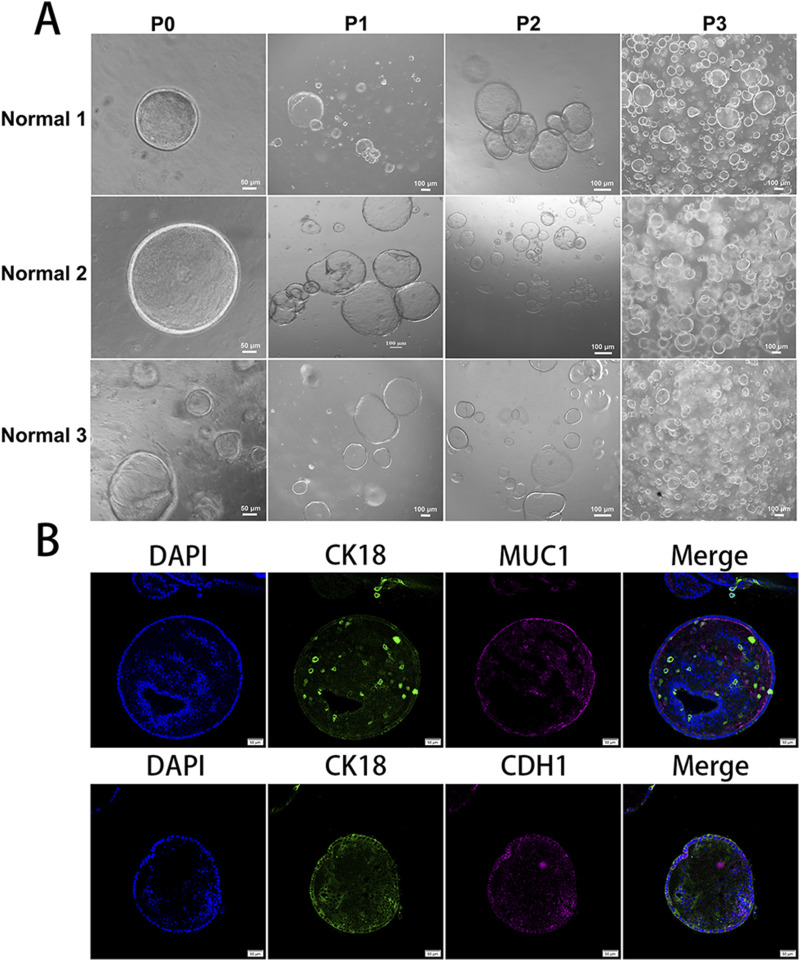
Establishment of endometrial organoids: **(A)** representative endometrial organoids obtained from three patients from passage 0 (P0) to passage 3 (P3); the scale bars for P0 are 50 μm, and the scale bars for P1 to P3 are 100 μm. **(B)** Immunofluorescence staining of organoids for CK18, MUC1, and CDH1 in fixed endometrial organoids or in thin sections (scale bar = 50 µm). Experiments were repeated twice.

Under appropriate conditions, fragmented endometrial tissue can self-assemble, leading to the formation of vesicular cystic structures within 7–10 days (P0). These primary organoids (P0) were subsequently dissociated and re-cultured across multiple passages (P1–P3), during which they consistently reformed and expanded. Furthermore, cryopreserved organoids were successfully revived and propagated. Representative bright-field microscopy images of this process are presented in Figure 1A. To confirm the components of the endometrial glandular cells, key biomarkers were identified using immunofluorescence analysis of fixed organoids and thin tissue sections. These included markers of epithelial cells (E-cadherin) and mucosal secretory cells (MUC1). Fluorescent signals corresponding to these markers were observed (Figure 1B), confirming that the EOs possess the fundamental cell types that are critical for replication and expansion.

### 3.2 Characteristics of endometrial organoids exposed to high androgens

**FIGURE 2:**
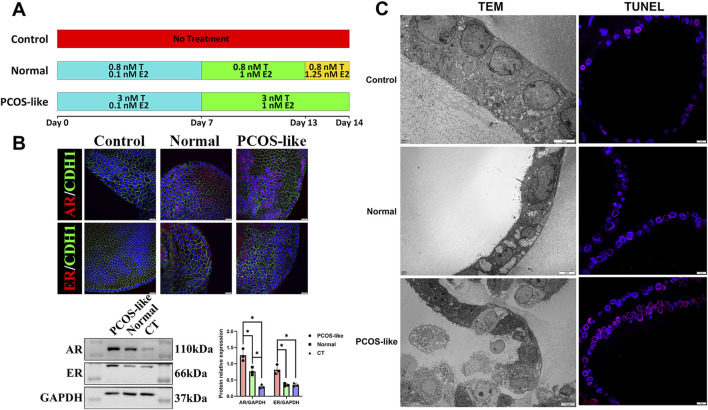
Hormonal effects on organoid morphological character and structure: **(A)** endometrial organoids’ hormone stimulation strategy. **(B)** Western blots and immunofluorescence staining of organoids treated in different ways for AR and ER (scale bar = 20 µm). * means *P* < 0.05. **(C)** Characteristics of endometrial organoids exposed to different treatments by TEM and TUNEL staining (scale bar: *left panel: upper = 5 µm*, *middle = 5µm*, and *bottom = 10µm; right panel: 10 µm*).

In organoids subjected to the 14-day stepwise E2 and T treatment (Figure 2A), the protein expression of the AR and ER was significantly higher than that in both the normal and control groups (Figure 2B), as determined by Western blot and immunofluorescence. Moreover, it was observed through TEM that compared to the control and normal groups (which exhibited visible microvilli, clear nuclei, abundant intracellular organelles, visible mitochondria, and endoplasmic reticulum), the PCOS-like group had sparse epithelial microvilli, small nuclei, shallower cytoplasm, significantly increased intracellular vesicles, and cell fragmentation, along with significantly increased TUNEL signaling confirmed by the TEM results (Figure 2C), indicating that the high levels of androgens have a damaging effect on the cells of the EOs.

### 3.3 Quantitative metabolomics analysis of the EOF

**FIGURE 3:**
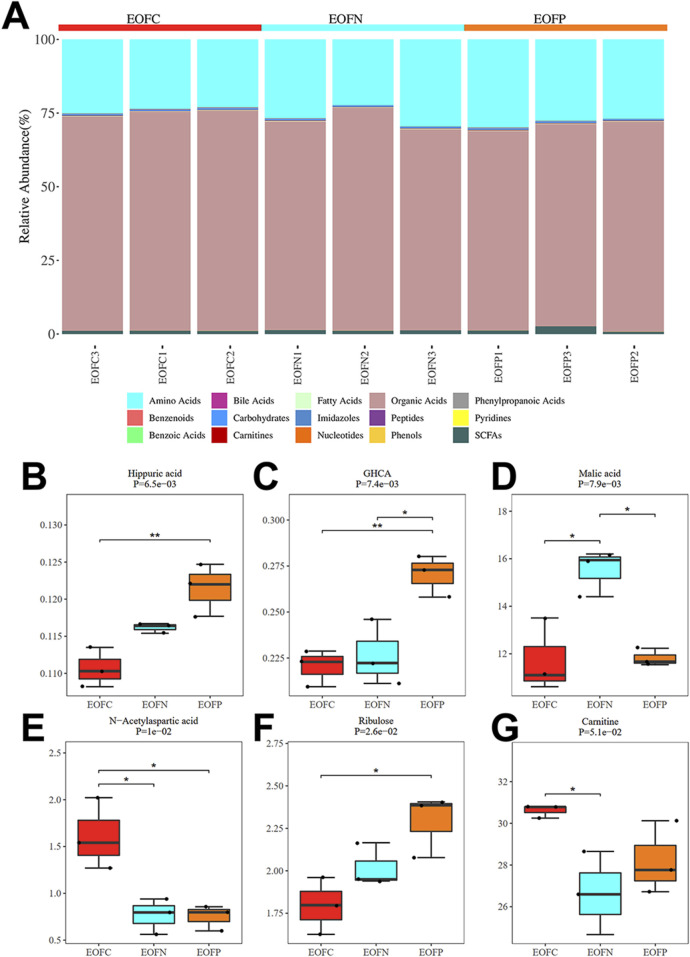
Composition of the EOF and the significantly changed metabolites: **(A)** relative abundance of major metabolite classification for the entire metabolomic profiles in the EOF. **(B-G)** Six significantly changed metabolites among the EOFC, EOFN, and EOFP groups.

The above results indicate that high levels of androgens damage organoid cells. To further understand the effect of excessive androgen levels on the metabolites of uterine cells and to provide a better reference for clinical treatment, the EOF, which is a quasi-representative model of the uterine epithelial–stromal cell interface and underlying vasculature, was used (Simintiras et al., 2021). High-throughput untargeted ultrahigh-performance liquid chromatography (LC)-MS was performed to detect metabolomic differences between the EOF of the control group (EOFC), the normal group (EOFN), and the PCOS-like group (EOFP). The results shown in Figure 3A indicate that 137 metabolites were identified in the EOF, including amino acids, bile acids, fatty acids, organic acids, phenylpropanoic acids, benzenoids, carbohydrates, imidazoles, peptides, pyridines, benzoic acids, carnitines, nucleotides, phenols, and short-chain fatty acids (SCFAs). Organic acids accounted for 71.72% of the metabolites, while amino acids accounted for 25.9%. These two classes of metabolites represented the largest proportions. The metabolites with significant changes between the EOFC/EOFN/EOFP groups mainly include hippuric acid (benzoic acid), GHCA (bile acid), malic acid (organic acid), N-acetylaspartic acid (amino acid), ribulose (carbohydrate), and carnitine (Figures 3B–G).

Specifically, the metabolomic components between EOFC and EOFN (Supplementary Figure S1A), compared with those in the Control group Four metabolites showed significant changes (Supplementary Figures S1B-E): malic acid (organic acid, P = 0.023), hippuric acid (benzoic acid, P = 0.026), N-acetylaspartic acid (amino acid, P = 0.026), and fructose (carbohydrate, P = 0.048). Among these, the first two were significantly upregulated, and the latter two were significantly downregulated. The enriched signaling pathways (Supplementary Figure S1F) mainly include alanine, aspartate, glutamate metabolism, and the citrate cycle (TCA cycle) [-ln (p) > 3].

The metabolomic components of the EOFP group were compared with those in the control group (Supplementary Figure S2A). Six metabolites had significant changes (Supplementary Figure S2B-G): GHCA (bile acid, P = 0.0045), hippuric acid (benzoic acid, P = 0.013), N-acetylglutamine (amino acid, P = 0.015), N-acetylaspartic acid (amino acid, P = 0.021), ribulose (carbohydrate, P = 0.026), and GCA (bile acid, P = 0.047). Among them, GHCA, hippuric acid, N-acetylglutamine, and ribulose were significantly upregulated, whereas N-acetylaspartic acid and GCA were significantly downregulated. The concentrated signaling pathways mainly include alanine, aspartate, and glutamate metabolism (Supplementary Figure S2H).

The metabolomic components of the EOFP group were compared with those of the EOFN group (Supplementary Figure S3A). Six metabolites showed significant changes (Supplementary Figures S3B-G): malic acid (organic acid, P = 0.0035), N-acetylserine (amino acid, P = 0.0092), maleic acid (organic acid, P = 0.013), GHCA (bile acid, P = 0.022), glutamine (amino acid, P = 0.025), and glycylproline (peptide, P = 0.026). Malic acid and glutamine were significantly upregulated, and N-acetyl aspartic acid, N-acetyl serine, maleic acid, and glycylproline were significantly downregulated. The enriched signaling pathways mainly include glutamate, alanine, aspartate metabolism, TCA cycle, and D-glutamine and D-glutamate metabolism (Supplementary Figure S3H).

In general, changes in metabolites in the extracellular fluid of EOs in the PCOS-like group mainly focused on amino acid and energy metabolism, primarily alanine, aspartate, and glutamate metabolism.

### 3.4 Quantitative metabolomic analysis of the IOF

**FIGURE 4:**
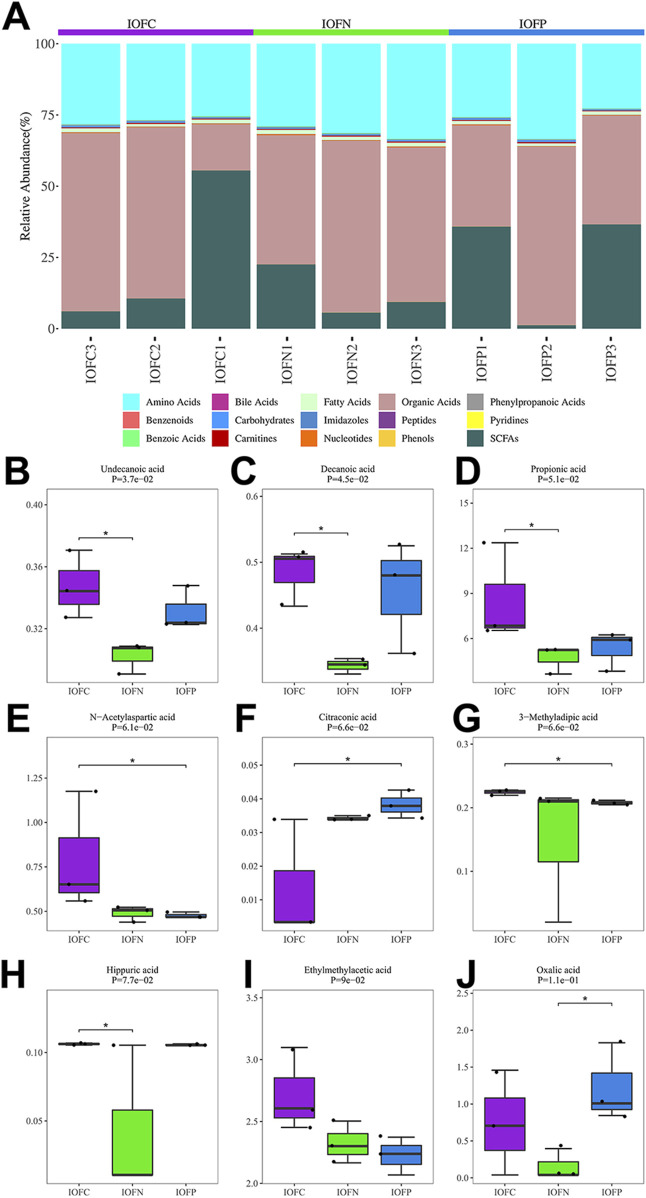
Composition of the IOF and the significantly changed metabolites: **(A)** relative abundance of major metabolite classification for the entire metabolomic profiles in the IOF. **(B-J)** Significantly changed metabolites among the IOFC, IOFN, and IOFP groups.

The IOF was representative of the uterine lumen (Simintiras et al., 2021). In the same way, metabolomic differences were analyzed among the IOFs of the control (IOFC), normal (IOFN), and PCOS-like (IOFP) groups, in addition to the EOFs of the control (EOF C), normal (EOF N), and PCOS-like (EOF P) groups. Figure 4A shows that 137 metabolites, including amino acids, bile acids, fatty acids, organic acids, phenylpropionic acid, benzenoids, carbohydrates, imidazoles, peptides, pyridines, benzoic acids, carnitines, nucleotides, phenols, and SCFAs, were identified in the IOF of EOs. Organic acids accounted for 50.9% of the metabolites, amino acids accounted for 28.69%, and SCFAs accounted for 17.63%. There were significant changes in metabolites between the IOFC/IOFN/IOFP groups, including undecanoic acid (fatty acid), decanoic acid (fatty acid), propionic acid (organic acid), N-acetylaspartic acid (amino acid), citraconic acid (organic acid), 3-methyladipic acid (fatty acid), hippuric acid (benzoic acid), ethylmethylacetic acid, and oxalic acid (Figures 4B–J).

Moreover, the heatmap displayed all metabolites in the IOFN vs. IOFC comparison (see Supplementary Figure S4A), the IOFC vs. IOFP comparison (see Supplementary Figure S4E), and the IOFN vs. IOFP comparison (see Supplementary Figure S4F). Three metabolites showed significant changes between the IOFN and IOFC groups: decanoic acid (fatty acid, P = 0.0058), palmitoyl carnitine (carnitine, P = 0.023), and undecanoic acid (fatty acid, P = 0.032), and all three metabolites were significantly reduced in the IOFN group (Supplementary Figures S4B-D). Only one significantly reduced metabolite, 3-methyl adipic acid (fatty acid, P = 0.0066), was detected in the IOFP group compared to the IOFC group (Supplementary Figure S4G). Similarly, undecanoic acid (fatty acid, P = 0.044) was the only significantly increased metabolite in the IOFP group compared to the IOFN group (Supplementary Figure S4H). No prominent signaling pathways were identified between the IOFN and IOFC groups, the IOFC and IOFP groups, and the IOFN and IOFP groups. In summary, the primary change in metabolites was in the fatty acids in the IOF of the PCOS-like group.

### 3.5 *In vitro* model confirms glutamine-enhanced endometrial stromal cell decidualization with altered mitochondrial function during the implantation process

**FIGURE 5:**
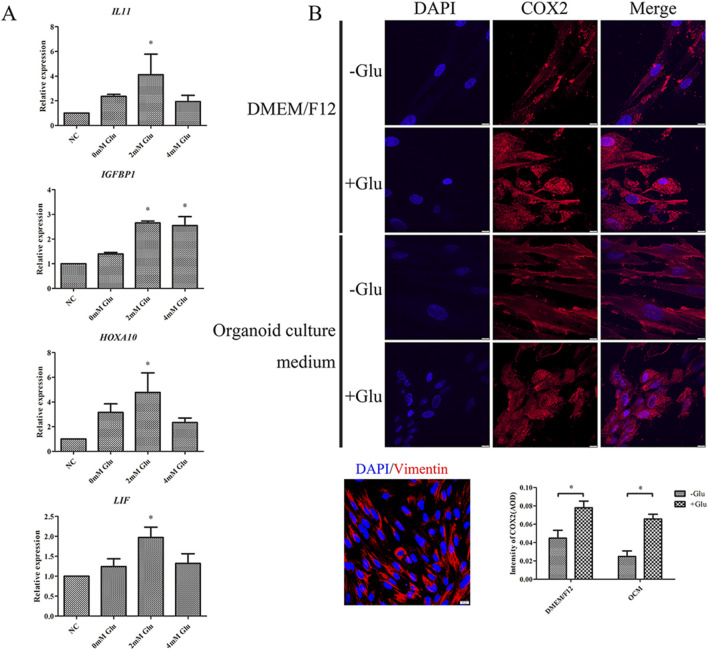
Decidualization level of hESCs was affected by the concentration of glutamine: **(A)** 2 mM of glutamine significantly increased IL11, IGFBP1, HOXA10, and LIF expression and accelerated hESC decidualization (P < 0.05). **(B)** The addition of 2 mM glutamine accelerated the COX2 level and hESC decidualization (P < 0.05; scale bar = 20 μm).

The interaction between epithelial cells and stromal cells plays a crucial role in the establishment of human endometrial receptivity. As demonstrated earlier, glutamine levels were significantly elevated in the EOFP group (Supplementary Figure S3H), indicating that glutamine may contribute to stromal cell decidualization. To explore this further, we tested the effects of different glutamine concentrations on stromal cell decidualization. Based on the relative expression levels of marker genes such as IL-11, IGFBP1, LIF, and HOXA10, indicated that an additional 2 mM of glutamine was the optimal concentration to enhance decidualization. However, higher glutamine levels showed no significant difference compared to 0 mM or the NC group (Figure 5A). Immunostaining for COX2 in the 2 mM group revealed a marked increase in COX2 expression compared with the glutamine-deficient group, whereas similar results were observed in the conditional group (Figure 5B). These findings indicate that supplementing with an additional 2 mM of glutamine promotes endometrial stromal cell decidualization.

Glutamine also functions as an energy substrate for mitochondrial metabolism, which was significantly enriched in the pathway analysis (Supplementary Figure S1F). We hypothesized that it might influence decidualization by modulating mitochondrial activity. To analyze this, we assessed changes in mitochondrial numbers using immunofluorescence. Our results showed no significant differences in TOMM20 and OPA1, indicating that mitochondrial numbers did not increase following the additional glutamine supplementation (Supplementary Figure S5A). However, RT-qPCR analysis showed partial upregulation of genes involved in mitochondrial fusion and fission, whereas others remained unchanged (Supplementary Figure S5B). These findings indicate that glutamine accelerates stromal cell decidualization, at least partially, by enhancing mitochondrial activity.

**FIGURE 6:**
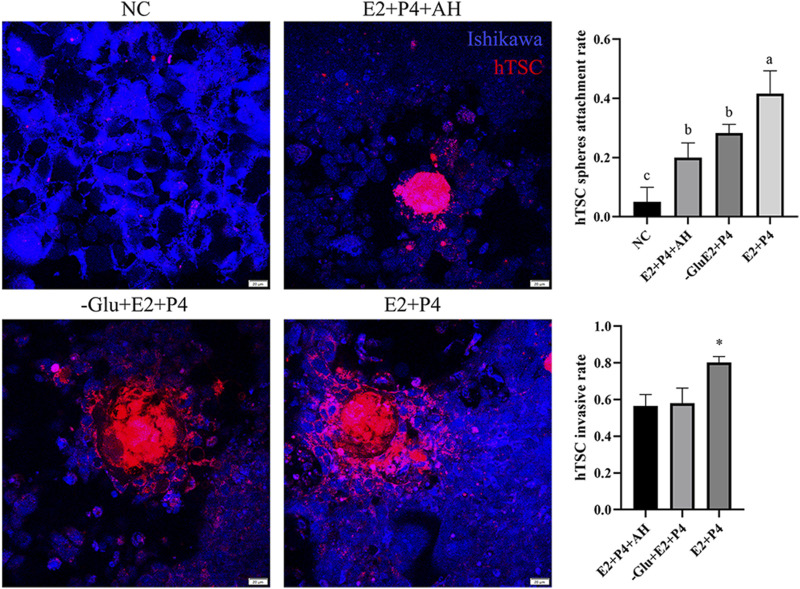
Optimal glutamine is necessary for embryo adhesion. Compared to the estrogen and progesterone stimulation group, the androgen addition group, and the group lacking glutamine, the hTSC sphere adhesion and invasion rates were reduced. Red cell tracker-labeled hTSC spheres and blue cell tracker-labeled Ishikawa cell layers are shown. a, b, and c indicate P < 0.01; * indicates P < 0.01.

Additionally, we examined whether glutamine deficiency or high levels of androgen exposure affect embryo attachment. Using hTSC spheres as embryo models, we evaluated the adhesion behavior of Ishikawa cell layers treated with extra glutamine for 5 days (Figure 6). As expected, the group treated with estrogen and progesterone achieved significantly higher adhesion rates than the hormone-free control group (0.417 ± 0.076 vs. 0.05 ± 0.05, P < 0.01). The groups treated with either extra androgen or glutamine deficiency showed no significant differences, but showed higher adhesion rates than the negative control group (0.2 ± 0.05 vs. 0.05 ± 0.05; 0.283 ± 0.029 vs. 0.05 ± 0.05, P < 0.01). Interestingly, the sphere invasion rate was lower in both the androgen-treated and glutamine-deficient groups than in the positive control group (0.802 ± 0.033 vs. 0.565 ± 0.063 and 0.579 ± 0.0836, P < 0.01). Taken together, our results show that glutamine has an essential function in stromal cell decidualization.

## 4 Discussion

In this study, we established a PCOS-like model derived from the EO model through androgen induction and found several novel observations regarding the metabolic characteristics and potential pathological implications in this model. Both immunofluorescence and EM staining demonstrated that the model can reflect the pathological changes similar to those in the *in vivo* condition. Second, using this model, we identified significant differences between the metabolites of the EOF and IOF, indicating that the active transport of metabolites along the endometrium is critical for molecular changes during the implantation process. Third, our results indicate distinct changes in metabolites with different characteristics in the EOF and IOF in the PCOS-like model compared to the normal control group (which received normal hormone treatment) and the control group (which did not receive hormone treatment). High androgen treatment leads to significant changes in major EOF metabolites, including those involved in amino acid and energy metabolism. Fatty acids, among other metabolites, are major molecules identified in the IOF and provide possible pathophysiological insight into environmental changes in the implantation window that are relevant to PCOS-related pregnancy (Supplementary Figure S6).

Our results indicate that glutamine metabolism and the TCA cycle are the most significantly enriched pathways altered by high androgen treatment, indicating that androgen treatment may lead to these metabolic changes in the uterine epithelial cells. The enrichment of glutamine metabolism and the TCA cycle in the EOF model is highly relevant to the PCOS-like environment, which may relate to implantation failure or miscarriage. A key characteristic of decidualized stromal cells is their high energy demand and their location in contact with the basal side of epithelial cells. The highly enriched glutamine metabolism implies that the glutamine transport is actively involved in the implantation process and the PCOS-like endometrium. Recent studies demonstrate that glutamine metabolites are upregulated in the endometrium of women with repeated miscarriages Hong et al., 2021), and reduced glutamate metabolism is closely linked to PCOS. Furthermore, a rat PCOS model has shown that glutamine supplementation can ameliorate inflammation and related phenotypes (Wu et al., 2020). Therefore, alterations in glutamine metabolism could have a critical function in the signaling pathway between epithelial and stromal cells during the implantation process. Nevertheless, our study is also limited by its small sample size. Another issue that should be considered is whether this PCOS-like model can reflect the real PCOS local metabolic changes, which need further study, as this study is a simplified hormonal model. Therefore, a larger sample size of both normal controls and PCOS patients is needed for further clarification of whether glutamine metabolism alterations are a general mechanism for PCOS-related metabolic changes. Understanding the exact metabolic changes in this pathway holds promise for future intervention strategies for the treatment of PCOS-related endometrial defects.

Our metabolic profiling of the major changed metabolites indicates that fatty acids are the major metabolites in the IOF of EOs in the PCOS-like group, which has several possible implications (Figure 3B). Embryo implantation is a dynamic process, and the IOF is the reflection of the environment directly involved in the interaction with the embryo. Therefore, glutamine-altered metabolites may impact the cross-talk of the embryo with the endometrium. First, the metabolic results agree with the EM results, which show that lipid accumulation is a significant feature of the PCOS-like model, and our metabolic results show that fatty acid changes are the most significantly enriched metabolites on the apical side of the endometrium, indicating an active fatty acid metabolism. Fatty acids are broadly involved in metabolic regulation, and their distribution is a key feature of metabolic syndrome associated with PCOS. It has been shown that PCOS causes a key change in ovarian fluid, and dysregulated fatty acids are a key metabolic change occurring in the uterine lumen of PCOS patients. A recent study has shown that PCOS patients have an evolutionary change in fatty acid deposition (Ma et al., 2022), supporting the hypothesis that fatty acid changes are closely associated with PCOS-related local receptive environment establishment.

Understanding the lumen and the outer region of the endometrium is key to understanding the intervention strategy for recurrent implantation failure (RIF) in PCOS patients. Our study indicates that targeting local glutamine metabolic changes may represent a novel strategy for repairing an injured endometrium. For example, our recent study used mesenchymal stem cell-derived material to rescue implantation failure (Lv et al., 2024). Emerging evidence shows that PCOS is a metabolic disease, and improving metabolic parameters can significantly change pregnancy outcomes. As free fatty acid levels, such as those of 3-methyl adipic acid, changed significantly in the PCOS-like model, the identified metabolic molecules warrant further confirmation in uterine fluid to identify whether they can be used as biomarkers and to design targeted interventions in cases of PCOS-related implantation failure or miscarriage.

## 5 Conclusion

In the present study, three cases of EOs derived from clinical endometrial samples were constructed successfully using optical microscopy and immunofluorescence. EOs with excess androgen were obtained by adding different ratios of estradiol and testosterone. Moreover, the morphology of the organoids and the expression of ER and AR were further detected by scanning electron microscopy and immunofluorescence. The IOF and EOF of the organoids were separated and sequenced using high-throughput quantitative metabolomics. The results confirmed differences in metabolites between the IOF and EOF. Amino acid metabolism and energy metabolites were the main metabolic pathways altered in EOF, whereas changes in fatty acids represented the main metabolite differences in IOF among the groups, which could be etiological factors of PCOS, as excess or lack of glutamine can lead to failed embryo implantation. This study provides opportunities to better understand how embryo implantation is affected by uterine fluid composition and the dynamic regulation of the endometrial epithelium in PCOS patients, which may provide metabolic marker screening and mechanistic insight into female fertility and offspring well-being.

## Data Availability

The raw data supporting the conclusions of this article will be made available by the authors, without undue reservation.
