# Supplementary figures and images for: Metabolic profiling of a polycystic ovary syndrome-like organoid model reveals the critical role of glutamine in local endometrial dysregulation related to implantation failure
Source: Front Cell Dev Biol. 2026 Mar 25;14:1751258. doi: 10.3389/fcell.2026.1751258 (PMC13056610; doi:10.3389/fcell.2026.1751258)

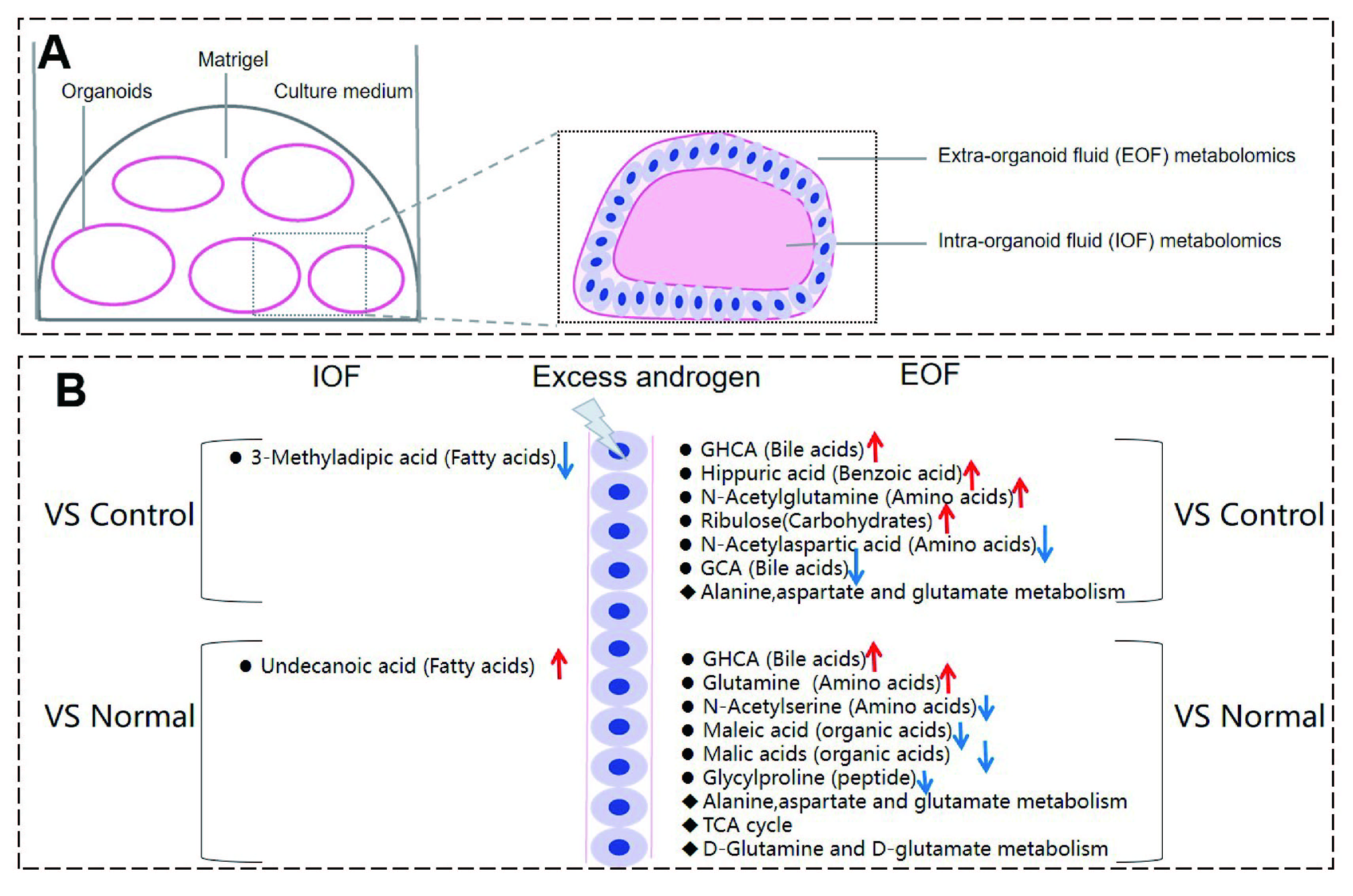

Supplement: Supplementary file 3 [file Image6.tif]

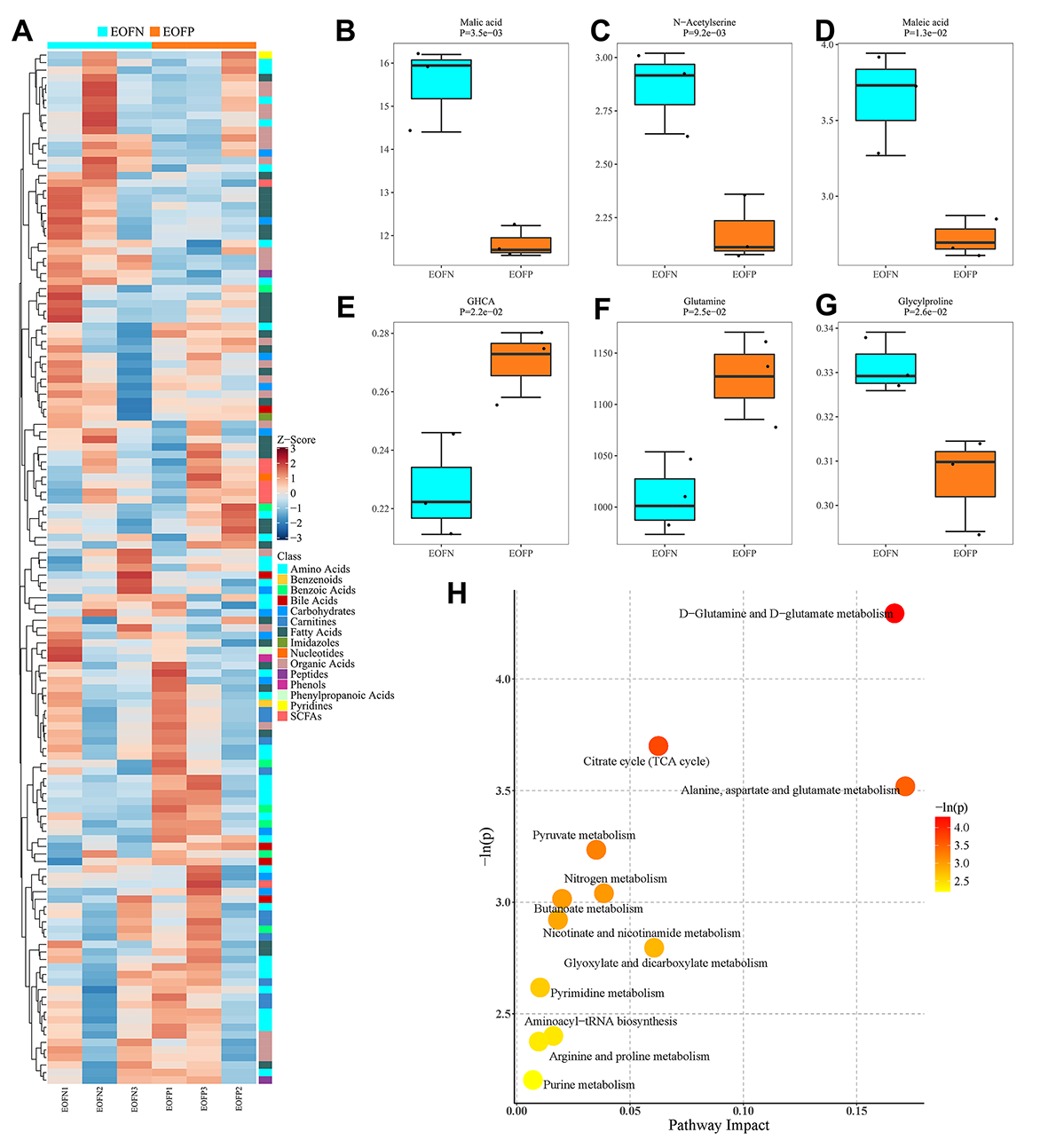

Supplement: Supplementary file 4 [file Image3.tif]

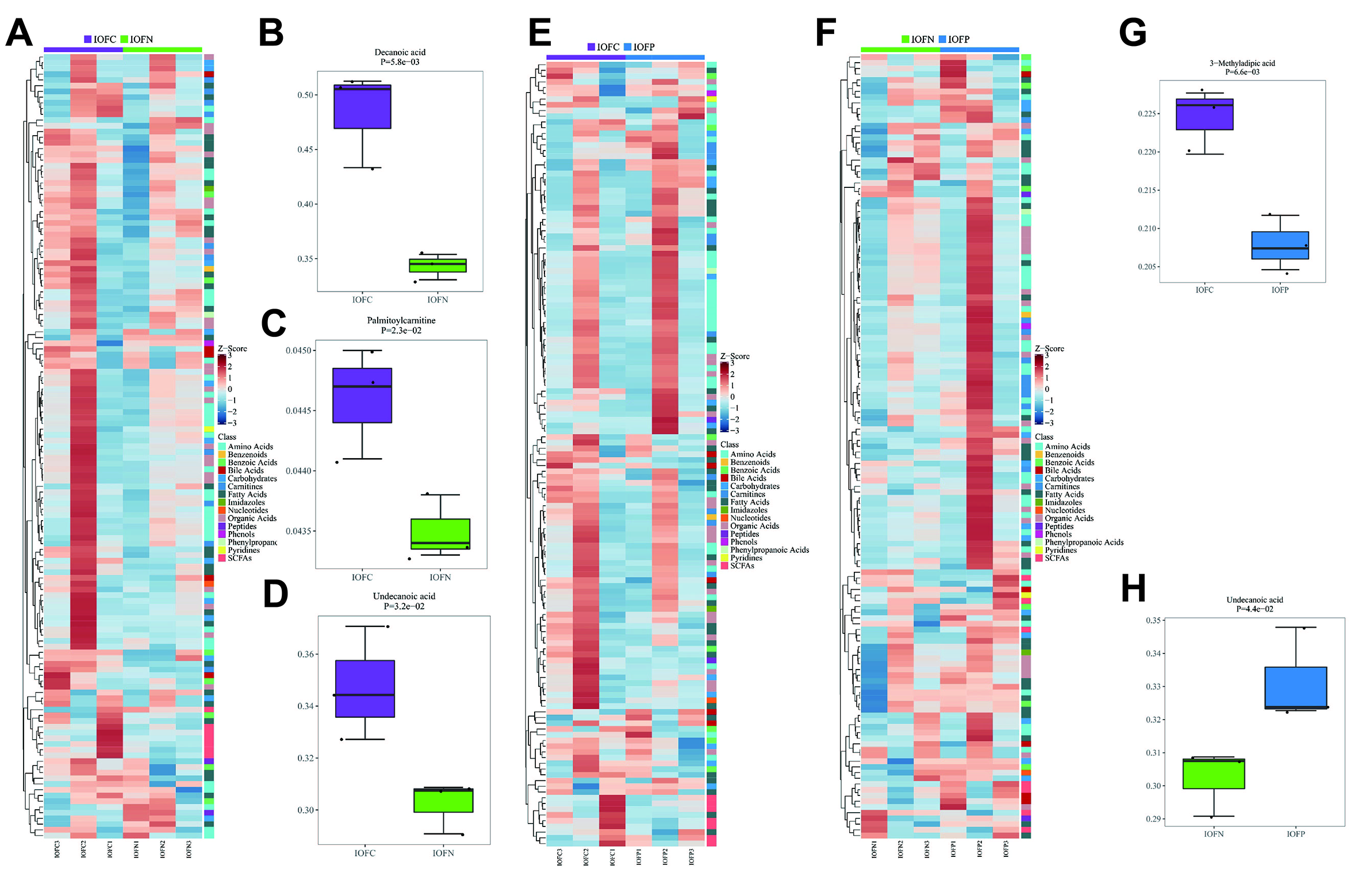

Supplement: Supplementary file 5 [file Image4.tif]

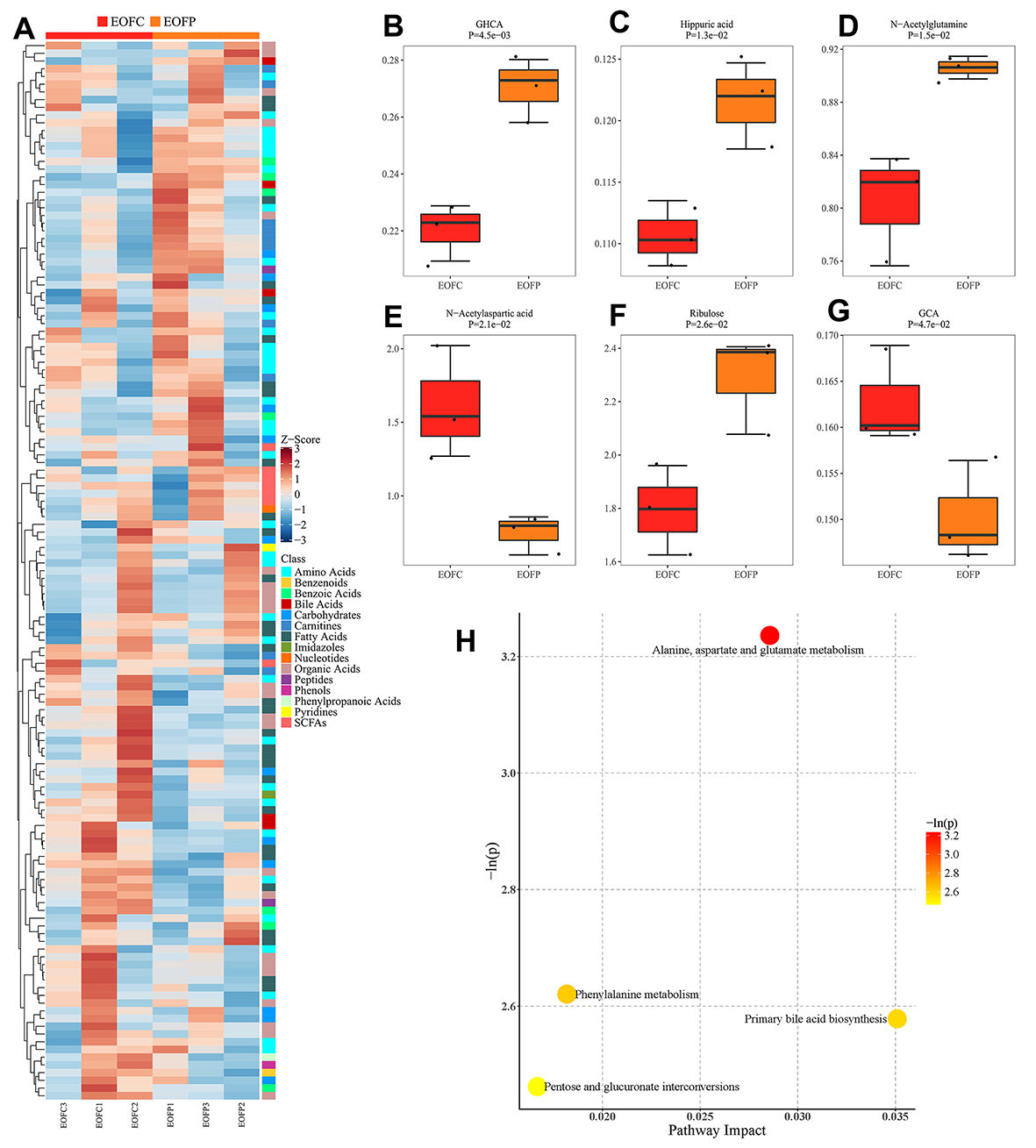

Supplement: Supplementary file 6 [file Image2.tif]

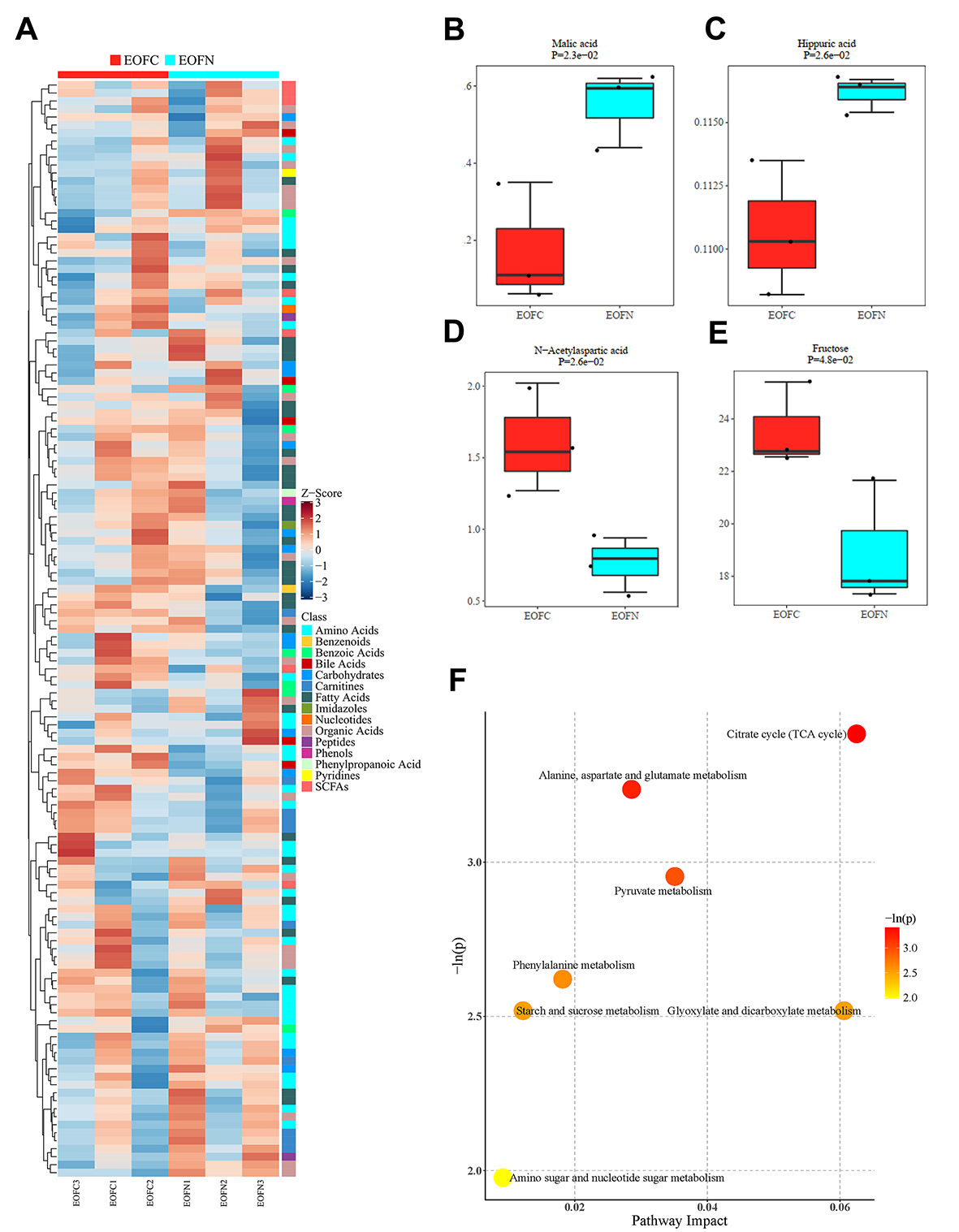

Supplement: Supplementary file 7 [file Image1.tif]

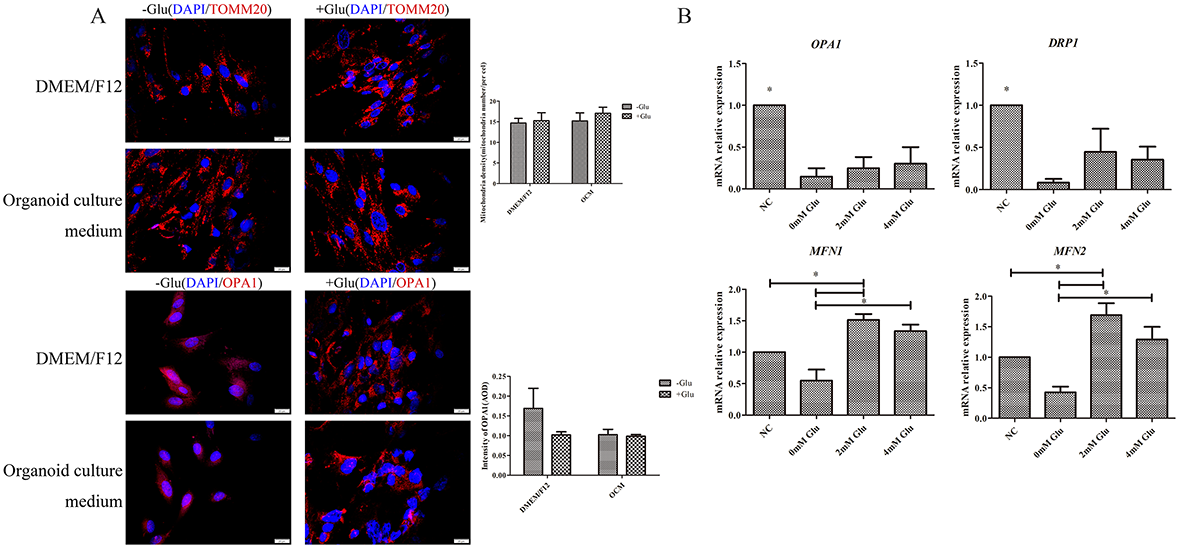

Supplement: Supplementary file 10 [file Image5.tif]
